# Paternal low protein diet and the supplementation of methyl-donors impact fetal growth and placental development in mice

**DOI:** 10.1016/j.placenta.2020.10.020

**Published:** 2021-01-01

**Authors:** Hannah L. Morgan, Arwa Aljumah, Charlène Rouillon, Adam J. Watkins

**Affiliations:** a Division of Child Health, Obstetrics and Gynaecology, Faculty of Medicine, University of Nottingham, Nottingham, NG7 2UH, UK; b INRAE, Fish Physiology and Genomics, Bat 16A, Campus de Beaulieu, Rennes, France; c Aston Research Centre for Healthy Ageing, School of Life and Health Sciences, Aston University, Birmingham, B4 7ET, UK^1^

**Keywords:** Paternal diet, Methyl donor supplementation, Fetal growth, Placental morphology, Developmental programming, Mouse model

## Abstract

**Introduction:**

Paternal low-protein diet can alter sperm methylation status, fetal growth and program offspring ill-health, however its impact on the placenta remains poorly defined. Here we examine the influence paternal low-protein diet has on fetal and placental development and the additional impact of supplementary methyl-donors on fetoplacental physiology.

**Methods:**

Male C57BL/6J mice were fed a control normal protein diet (NPD; 18% protein), a low-protein diet (LPD; 9% protein) or LPD with methyl-donor supplementation (MD-LPD; choline chloride, betaine, methionine, folic acid, vitamin B12) for a minimum of 8 weeks. Males were mated with 8–11 week old female C57BL/6J mice and fetal and placental tissue collected on embryonic day 17.5.

**Results:**

Paternal LPD was associated with increased fetal weights compared to NPD and MD-LPD with 22% fetuses being above the 90th centile for fetal weight. However, LPD and MD-LPD placental weights were reduced when compared to NPD. Placentas from LPD fathers demonstrated a reduced junctional zone area and reduced free-fatty acid content. MD-LPD placentas did not mirror these finding, demonstrating an increased chorion area, a reduction in junctional-specific glycogen staining and reduced placental *Dnmt3b*expression, none of which were apparent in either NPD or LPD placentas.

**Discussion:**

A sub-optimal paternal diet can influence fetal growth and placental development, and dietary methyl-donor supplementation alters placental morphology and gene expression differentially to that observed with LPD alone. Understanding how paternal diet and micro-nutrient supplementation influence placental development is crucial for determining connections between paternal well-being and future offspring health.

## Highlights

• Paternal low protein diet (LPD) increased late gestation fetal weight.
• Supplementing the LPD with methyl donors (MD-LPD) normalised fetal weight.
• Placental weight and morphology are altered by both LPD and MD-LPD.
• Placental metabolite content and gene expression were perturbed by paternal LPD and MD-LPD.

## 1 Introduction

Placental dysfunction can cause extremes in fetal growth, such as the development of small for gestational age (SGA) babies (defined as a fetal weight under the 10th percentile for the population at that gestational age) or fetal overgrowth (a fetal weight over the 90th percentile, termed large for gestational age (LGA)) [1,2]. These abnormalities in fetal growth are highly prevalent worldwide, and have been linked to an increased risk of stillbirth and childhood morbidities [[3], [4], [5]]. There is also evidence that inappropriate growth *in utero* can impact childhood development and offspring health in adulthood [6]. David Barker and colleagues' extensive studies of epidemiological data developed the ‘fetal programming’ hypothesis, and first demonstrated associations between maternal nutrition, impaired fetal growth and the risk of developing cardiovascular or metabolic disorders in later life [[7], [8], [9]]. This hypothesis suggests exposure to sub-optimal environments during crucial developmental time-points can alter fetal growth trajectories, predisposing the offspring to increased risk of poor health in later life [6].

A range of human and animal model studies have explored the role of maternal diet during gestation on placental and fetal development [10,11]. More recently, studies have identified the pre-conception period as a critically sensitive time during which adverse environmental conditions can affect fetal development and offspring health [12]. In mice, a maternal low-protein diet fed exclusively during oocyte maturation altered offspring behavioural phenotype and increased their susceptibility to cardiovascular disease [13,14]. Similar findings have been reported in sheep, where maternal peri-conceptional caloric restriction resulted in cardiovascular abnormalities in the lambs, including increased heart rate and blood pressure [15]. Maternal peri-conceptional folate, vitamin B12 and methionine restrictions in sheep have also been shown to reduce fetal liver DNA methylation levels and program increased adiposity and cardiovascular dysfunction in the offspring [16]. As our understanding of the sensitivity of the pre- and peri-conception period expands, a new focus on the role the father's diet has for embryo development, implantation and fetal growth is emerging.

We have previously shown that a paternal sub-optimal low-protein diet (LPD) in mice resulted in increased adiposity, vascular dysfunction and impaired glucose tolerance in his offspring [17]. We also observed significant changes in expression of genes involved in metabolism, transcription and signalling cascades in the blastocyst [18]. In addition, we found that paternal LPD programmed fetal overgrowth and altered expression of placental transport, DNA methylation and imprinted genes, predisposing these offspring to cardiovascular and metabolic disorders in adulthood [17,18]. Paternal LPD has also been associated with a reduction in testicular DNA methyltransferase gene expression and changes in expression of genes involved in the 1-carbon metabolism pathway [19,20], alongside a global hypomethylation of their sperm [19]. A separate study examining reduced paternal folate intake in mice, found this dietary regime resulted in fetal craniofacial abnormalities and limb underdevelopment, which were associated with sperm DNA methylation status [21]. These studies not only show that paternal diet can influence embryo development but that his diet can influence the epigenetic status of the fetus, thus influencing developmental programming of the offspring [22,23]. However, there is a paucity of information regarding the impact paternal health has on the placenta. This study aimed to determine how paternal LPD in mice influences the placenta, with further investigations examining whether methyl-donor dietary interventions ameliorate any observed impairments to fetal or placental development, due to the intimate involvement of the 1-carbon metabolism in regulating epigenetic signatures.

## 2 Materials and methods

### 2.1 Animals and diet regimen

All animal procedures were approved by the Home Office according to the Animals (Scientific Procedures) Act 1986 (Project License 30/3253) and local ethical approval at Aston University. All C57BL/6J mice (Charles River, UK) were housed in controlled 12/12-h light/dark conditions with a constant temperature (21 °C ± 3 °C) and ad libitum access to water. Virgin 8-week old males were fed either control normal protein diet (NPD; 18% casein; n = 7), isocaloric low protein diet (LPD; 9% casein; n = 7), or isocaloric LPD supplemented with methyl-donors [an addition of 5 g/kg diet choline chloride, 15 g/kg diet betaine, 7.5 g/kg diet methionine, 15 mg/kg diet folic acid, 1.5 mg/kg diet vitamin B12 to LPD (MD-LPD; n = 6)] for a minimum of 8 weeks to ensure all stages of spermatogenesis were exposed to the diets, as previously described [20]. Dietary components are outlined in Supplementary Table 1.

Virgin female C57BL/6J mice were mated at 9-weeks old (±7 days) with stud males from one of the three diet groups (NPD; n = 7, LPD; n = 7, MD-LPD; n = 6). Successful mating was confirmed by the presence of a copulation plug, denoted as embryonic day (E)0.5. The dams were fed and maintained on standard rodent chow (rat/mouse No.1 maintenance diet, Special Diet Services), for the remainder of gestation. At E17.5 dams were euthanized via cervical dislocation and fetal and placental weights recorded. Placentas were collected from the middle two fetuses within each uterine horn (where possible); one snap-frozen and stored at −80 °C for molecular investigations and the other fixed in 10% neutral buffered formalin (Sigma; UK) at room temperature overnight, stored in 70% ethanol and processed for histology on a Thermo-Scientific Excelsior tissue processor.

### 2.2 Analysis of placental morphology and glycogen

Formalin fixed-paraffin embedded placental tissue was sectioned at 5 μm through the mid-line using a Leitz 1512 rotary microtome (Leica). Placental sections were stained with haematoxylin and eosin to assess gross morphological structure and layer proportions. Placental glycogen was stained using a periodic acid Schiff (PAS) stain. Briefly, sections were dewaxed and rehydrated before incubation with 0.5% periodic acid (Sigma, UK) for 90 min. Sections were then washed and incubated in Schiff's reagent (Sigma, UK) for 15 min, before dehydrating and mounting in DPX. The percentage of positive magenta staining was measured by a blinded operator using Image J software.

Total placental glycogen content was determined using the protocols of Lo et al. (1970) and Rampon et al. (2008) with some modifications [24,25]. Briefly, 10 mg of placental tissue was homogenised in 30% KOH saturated with Na_2_SO_4_ (Sigma, UK) and incubated at 100 °C for 30 min, followed by cooling on ice. Glycogen was precipitated in 100% ethanol and separated by centrifugation at 1,000×*g* for 30 min. Glycogen standards were prepared using Rabbit Liver Glycogen (G8876; Sigma, UK), with concentrations ranging from 400 μg/ml to 12.5 μg/ml. Samples and standards were combined with 5% phenol with H_2_SO_4_ (Sigma, UK) to initiate a colorimetric change. Absorbance was read at 490 nm on Benchmark Bio-Rad microplate reader.

### 2.3 Free fatty acid profile

Total placental free fatty acids (FFA) were quantified using the commercially available free fatty acid assay kit (ab65341, Abcam, UK). The extraction, preparation and colorimetric measurements of samples and standards was performed according to manufacturer's instructions. Briefly, 10 mg of placental tissue was homogenised in 1% TritonX-100 in chloroform (Sigma, UK), using Qiagen TissueLyserII (25 Hz, 2 × 30 s), centrifuged at 17,000×*g* at 4 °C for 10 min and the organic phase dried and re-constituted in assay buffer. Sample and standard absorbance were read at 570 nm on Benchmark Bio-Rad microplate reader. Concentrations were interpolated from the standard curve after blank subtraction and corrected for dilution, volume of sample and placental weight.

### 2.4 Cholesterol content assay

Placental lysate was obtained from 10 mg placental tissue in chloroform as outlined above. The cholesterol content of the dried lipids was determined using the Amplex-Red Cholesterol Assay Kit (A12216, Invitrogen), following manufacturer's instructions. Absorbance was read at 540 nm on Benchmark Bio-Rad microplate reader. Concentrations were interpolated from the standard curve after blank subtraction and corrected for dilution and weight of placental tissue.

### 2.5 Placental RNA extraction and gene expression

Placental RNA was extracted from 20 to 25 mg of frozen placental tissue using the RNeasy mini kit (Qiagen, UK), following tissue disruption in Qiazol (Qiagen, UK) using the Qiagen TissueLyserII (25 Hz, 4 × 30 s), according to manufacturer's instructions. RNA concentration was quantified by Nanodrop and cDNA templates produced using Precision nanoScript2 RT kit (PrimerDesign, UK) according to the manufacturer's instructions, with RNA input standardised for 1 μg. Real-time quantitative PCR was used to determine expression of key epigenetic and imprinted genes previously found to be dysregulated in the sperm and/or placenta in response to LPD [18,19], using 5 ng cDNA and 175 nM forward and reverse primers (Eurofins Genomics, Germany) with 1x Precision SYBRgreen Mastermix (PrimerDesign, UK) per reaction. All RT-qPCR was performed using an Applied Biosystems 7500 system. Gene expression was analysed using the delta-delta Ct method relative to NPD expression, the GeNorm method was used to normalise gene expression, as previously described [26], to two reference genes (*Tbp* and *Tuba*). Briefly the geometric mean of both reference genes was calculated for all samples, and for the total number of samples used, to construct a normalisation factor which was then used to normalise each samples' gene expression. Details of all primers can be found in Supplementary Table 2.

### 2.6 Placental DNA extraction and methylation quantification

Total placental DNA was extracted from 20 to 25 mg of placental tissue using the DNeasy Blood & Tissue Kit (Qiagen, UK), following tissue disruption in Buffer ATL (Qiagen, UK) using the Qiagen TissueLyserII (25 Hz, 4 × 30 s), according to manufacturer's instructions. DNA concentration was quantified by Nanodrop and samples diluted to 50 ng/μl. Global DNA methylation was quantified using the Methylated DNA Quantification Kit (ab117128, Abcam, UK) following manufacturer's instructions with plates read at 450 nm, 2 min from reaction end, on a Benchmark Bio-Rad microplate reader. The relative levels of 5-methylcytosine were expressed as a percentage compared to the in-kit methylated polynucleotide positive control.

### 2.7 Statistical analysis

Fetal and placental data were analysed using generalised linear mixed model analysis, factoring for dam, littersize and uterine position, using SPSS [27]. Percentiles for NPD data were calculated using the formula (*Z-critical* × *SD*) + *mean*; where Z-critical value was −1.2816 (10th centile) and 1.2816 (90th centile) and statistical significance was determined using Chi^2^-test. Analysis and graphical interpretations of all other data were performed using GraphPad Prism (v7.0). Where appropriate, normality was assessed using Shaprio-Wilk and Kolmogorov-Smirnov tests, in the case data was normally distributed, one-way ANOVA with Tukey's post-hoc test was performed, and where data were not normally distributed, Kruskall-Wallis test with Dunns multiple comparison test was performed. Significance was determined as p < 0.05.

## 3 Results

### 3.1 Maternal characteristics

**Fig. 1:**
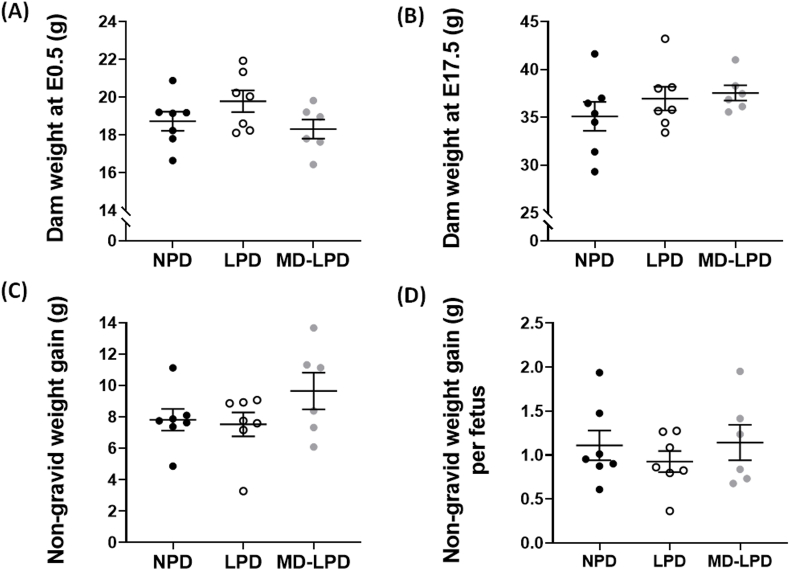
Maternal pregnancy associated weight gain. Maternal body weight at E0.5 (A), E17.5 (B), gestational weight gain independent of total conceptus mass (C) and mean weight gain per fetus (D). Data points represent individual dam, with mean ± SEM; statistical significance was determined using one-way ANOVA with Tukey post-hoc test, comparing the three diet groups; n = 6–7.

There was no significant difference in dam weight immediately following successful mating (Fig. 1A), at E17.5 (Fig. 1B) or after subtraction of total conceptus mass (non-gravid weight gain) (Fig. 1C). Additionally, no significant differences were observed in the maternal weight gain normalised for the number of fetuses (Fig. 1D).

### 3.2 Paternal diets impact on late gestation fetal and placental weights

**Fig. 2:**
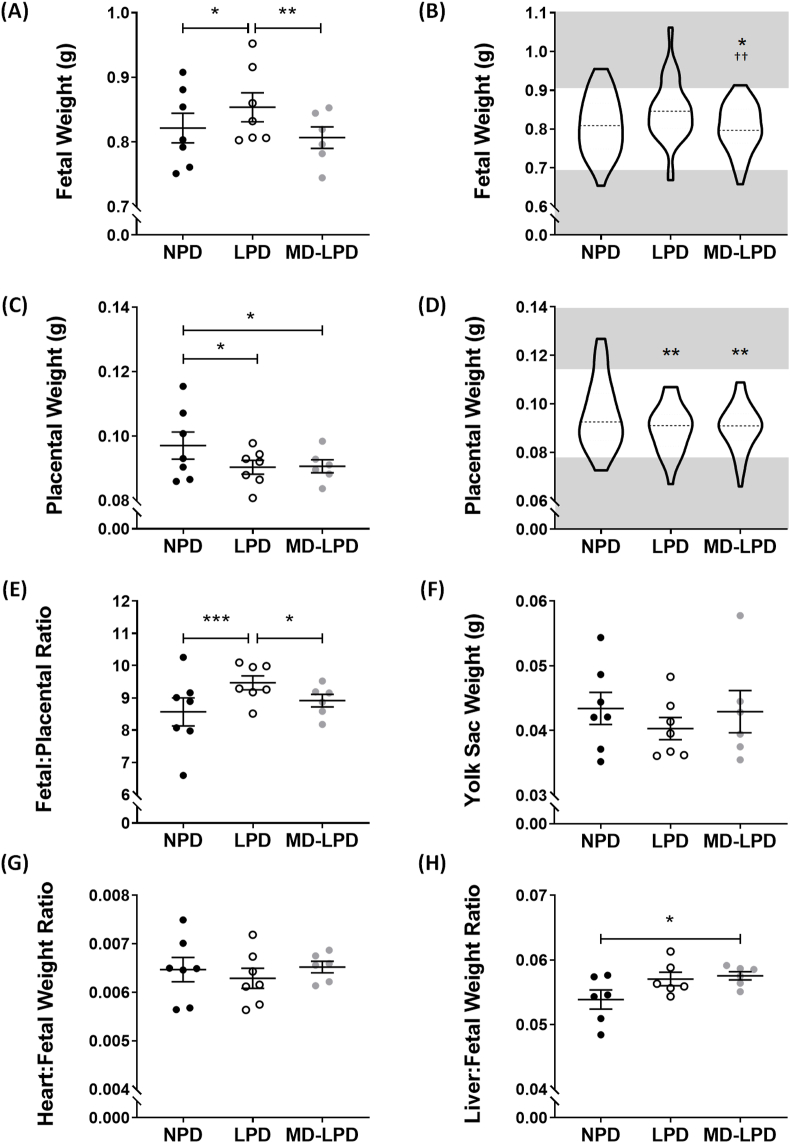
Paternal diet impacted fetal and placental parameters at E17.5. Fetal weights (A) and their distributions (B), placental weights (C) and their distributions (D), the fetal:placental weight ratio (E) and yolk sac (F). Fetal organs were dissected to allow calculations of the heart:fetal weight ratio (G) and liver:fetal weight ratio (H). Data presented as dot plot where each data point is average of entire litter, with line representation of mean ± SEM (N = 6–7 litters; n = 53–59 fetuses), statistical significance determined using a generalised linear mixed model analysis, *p < 0.05, **p < 0.01, ***p < 0.001; or as violin plot of all fetuses (n = 53–59) with the 10th and 90th centiles denoted by grey areas, significant differences in 90th centile proportions and 10th centile proportions calculated using Chi^2^ test, *p < 0.05, **p < 0.01 vs NPD and ^††^p < 0.01 vs LPD.

We have previously published that neither the paternal LPD, nor the supplemented MD-LPD, influence male reproductive success, as E17.5 litter size was not significantly altered when compared to NPD [20]. Fetuses sired from LPD fed fathers had a significantly increased weight at E17.5 compared to NPD (p = 0.0113) and MD-LPD (p = 0.0018) fetuses. (Fig. 2A). Fetal weight distributions revealed LPD fathers produced a higher proportion of ‘overgrown’ fetuses; 22% fall above the 90th centile for NPD fetal weights, however this did not reach significance when compared to NPD (Fig. 2B). In contrast, MD-LPD fetuses demonstrated a significantly reduced proportion of fetuses above this centile (1.9%) compared to NPD (p = 0.0294) and LPD (p = 0.0013) (Fig. 2B). Placental weights were found to be significantly reduced in both LPD (p = 0.0158) and MD-LPD (p = 0.0159) groups when compared to NPD placentas (Fig. 2C). The distributions of placental weights did not reflect the trends observed in the fetal weight distributions, and demonstrated a significant reduction in the proportion of placental weights above the 90th centile, with all placental weights in LPD (p = 0.0047) and MD-LPD (p = 0.0072) groups falling below this threshold (Fig. 2D). The fetal:placental ratio was examined as an indication of placental efficiency. We found that this ratio was significantly increased in LPD compared to NPD (p = 0.0005) and MD-LPD (p = 0.0386) groups (Fig. 2E). However, there was no significant difference in fetal:placental ratio between NPD and MD-LPD fetuses (Fig. 2E). There was no differences between diet groups in yolk sac weight, or heart:body weight ratio of the fetuses (Fig. 2F and G). Fetuses from MD-LPD fed fathers demonstrated significantly increased liver:body weight compared to NPD fetuses (p = 0.0412) (Fig. 2H).

### 3.3 Paternal diets impact on placental morphology

**Fig. 3:**
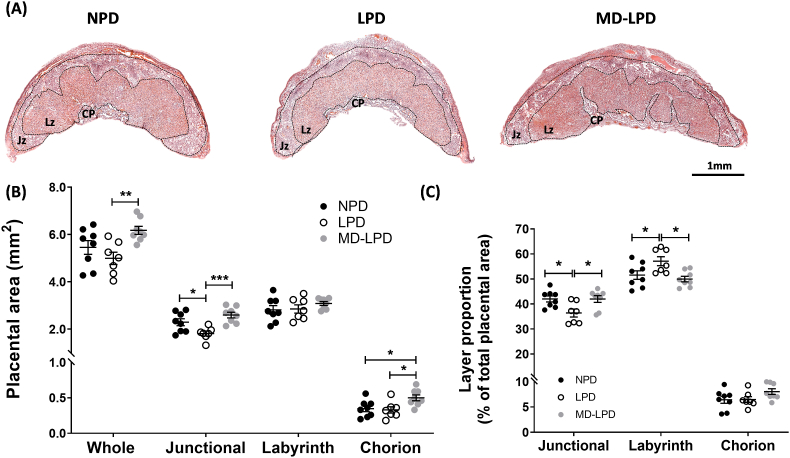
Paternal diet altered placental area. Representative images of H&E stained placenta with junctional (JZ) and labyrinth (LZ) zones and chorionic plate (CP) annotated (A). Measured placental area of whole placenta and respective zones (B) and the representative proportions of the three distinct zones (C). Each data point represents a single placenta, n = 4–6 litters (1–2 placenta assessed from each litter), data presented with mean ± SEM. Statistical significance was determined using one-way ANOVA for each zone with Tukey post-hoc test comparing the three diet groups; *p < 0.05, **p < 0.01, ***p < 0.001.

Representative images of H&E stained placental sections, highlighting the junctional, labyrinth and chorion zones, are shown in Fig. 3A. Overall, MD-LPD placentas had a significantly increased whole placental cross-section area compared to LPD (p = 0.0082), but no significant alterations were observed in LPD or MD-LPD when compared to NPD (Fig. 3B). LPD placentas did demonstrate a significant reduction in junctional area compared to NPD (p = 0.0383) and MD-LPD (p = 0.0009) placentas (Fig. 3B). MD-LPD placentas also demonstrated an expanded chorion area compared to both NPD (p = 0.0419) and LPD (p = 0.0284). The labyrinth area was the only zone area not to be impacted by any of the paternal diets (Fig. 3B). Upon examination of the layer proportions, we found placentas from LPD fathers had significantly reduced junctional zone percentage, and thus a proportionally increased labyrinth zone (p = 0.0275 vs NPD and p = 0.0265 vs MD—LPD) (Fig. 3C).

### 3.4 Placental glycogen content

**Fig. 4:**
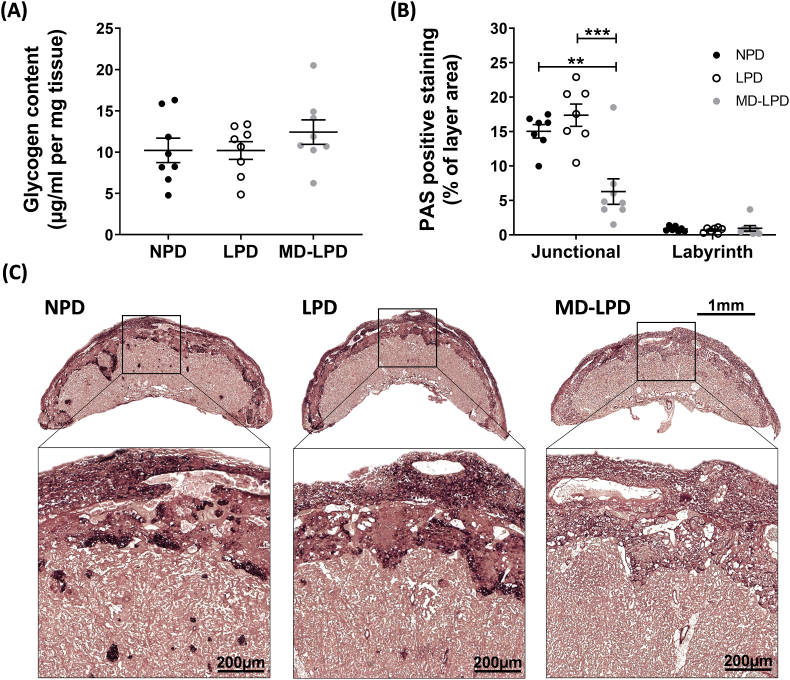
Assessment of paternal diet's impact on placental glycogen. Whole placental glycogen content (A) and positive PAS staining within placental zones, expressed as a proportion of total zone area (B). Representative images of PAS stained placental sections for each diet group, with a magnified view of positive junctional zone staining (C). Data presented as mean ± SEM, with each data point representing a single placenta; n = 4–6 litters (1–2 placenta assessed from each litter). Statistical significance was determined using one-way ANOVA with Tukey post-hoc test, comparing the three diet groups; **p < 0.01, ***p < 0.001.

Glycogen content of the placenta was analysed with a biochemical assay and PAS staining. The placental glycogen content of whole placental tissue was not significantly different between any of the diet groups (Fig. 4A). The PAS stained placenta revealed the majority of glycogen positive staining was localised to the junctional zone, and this was significantly reduced in MD-LPD placentas when compared to NPD (p = 0.0022) and LPD (p = 0.0002) (Fig. 4B and C).

### 3.5 Placental free-fatty acid and cholesterol content

**Fig. 5:**
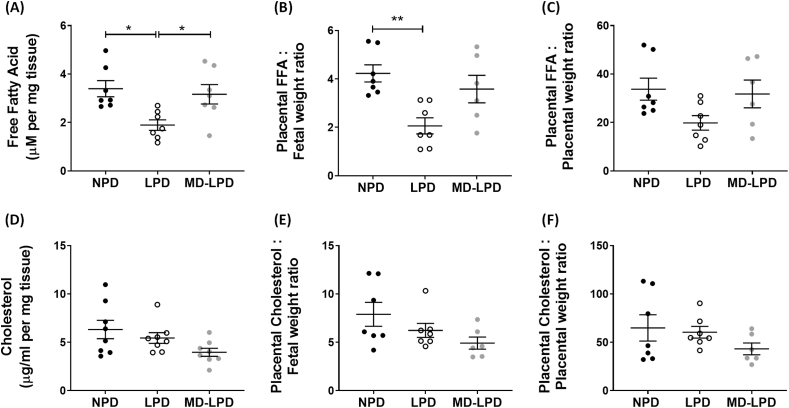
The impact of paternal diet on placental free fatty acid and cholesterol metabolism. Biochemical assay of E17.5 placental free fatty acid (FFA) (A), with the FFA content expressed as a proportion of fetal weight (B) and placental weight (C). Placental cholesterol content (D), expressed as a proportion of fetal weight (E) and placental weight (F). Data presented as mean ± SEM, with each data point representing a single placenta, n = 6–7 placentas (1 placenta/litter). Statistical significance was determined using one-way ANOVA with Tukey post-hoc test, comparing the three diet groups; *p < 0.05, **p < 0.05.

Placental free-fatty acid (FFA) concentrations were significantly reduced in whole placental extracts from LPD placenta compared to NPD (p = 0.0115) and MD-LPD (p = 0.0332) (Fig. 5A). The ratio of placental FFA and fetal weight was significantly reduced in the LPD group compared to NPD (p = 0.0041), with no significant differences observed in the MD-LPD ratio or in the FFA:placental weight ratio for any dietary groups (Fig. 5B and C). Paternal diet did not significantly alter placental cholesterol content per mg of placenta tissue (Fig. 5D), however there was a trend towards a reduced concentration in MD-LPD placentas (p = 0.0573). The ratios of placental cholesterol:fetal weight and cholesterol:placental weight were not significantly different between diet groups (Fig. 5E and F).

### 3.6 Placental expression of epigenetic regulatory and imprinted genes

**Fig. 6:**
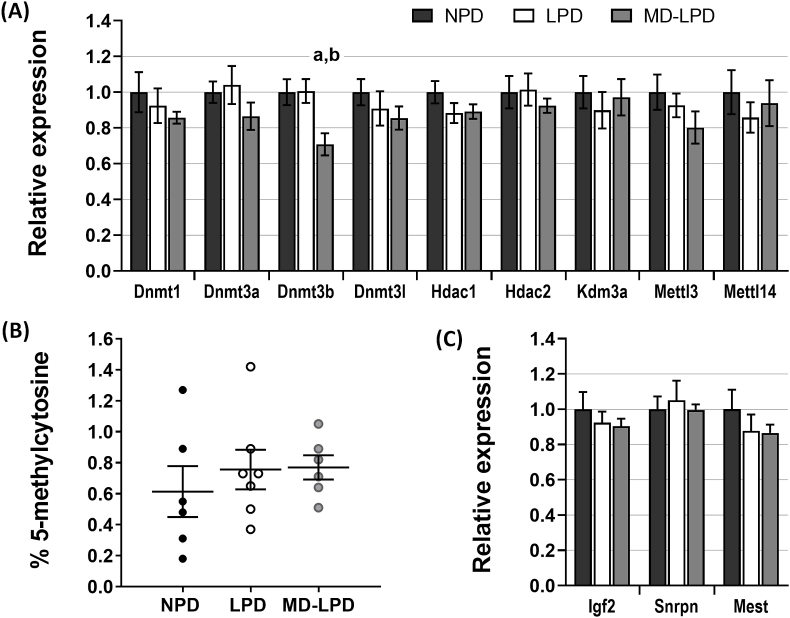
The placental expression of genes involved in epigenetic regulation (A) and the quantification of total placental 5-methylcytosine DNA methylation (B). Placental expression of three key paternally imprinted genes (C). All gene expression data presented relative to NPD expression normalised to *Tbp*and*Tuba*reference gene expression. n = 6–7 placentas (1 placenta/litter), statistical significance determined using either one-way ANOVA with Tukey post-hoc test or Kruskall-Wallis with Dunns post-test, comparing the three diet groups for each gene; a = p < 0.05 vs NPD, b = p < 0.01 vs LPD.

Of the investigated genes involved in epigenetic processes (Fig. 6A), MD-LPD placentas demonstrated a significantly reduced expression of *Dnmt3b* compared to NPD (p = 0.0212) and LPD (p = 0.0184) (Fig. 6A). However, there was no significant difference in the global proportion of methylated placental DNA (Fig. 6B). None of the three paternally expressed genes investigated demonstrated any significant changes in placental expression between the diet groups (Fig. 6C).

## 4 Discussion

There is increasing evidence that the father plays a prominent role in fetal programming, yet the mechanisms through which any sub-optimal paternal environment has on the development of his offspring is still relatively ill-defined. This study has provided evidence that paternal LPD increased late gestation fetal weight, yet was associated with a reduction in placental weight compared to NPD. Placentas from LPD fathers had a reduced junctional zone area and reduced free-fatty acid content, with no alteration in expression of key epigenetic and imprinted genes. This study also provided evidence that whilst supplemental dietary methyl-donors negate the observed LPD-directed fetal overgrowth, the placental phenotype did not mirror that of NPD or LPD groups. MD-LPD placentas exhibited a reduced weight, similar to LPD, yet did not demonstrate the LPD-associated proportional layer differences. MD-LPD placentas demonstrated unique findings; such as a reduced junctional specific glycogen content and reduced expression of *Dnmt3b*, the DNA methyltransferases gene involved in *de novo* methylation and increased chorion area. Thus, the supplementation of paternal LPD with methyl-donors did not prevent all LPD-directed changes to the placenta and exhibited a differentially altered structure. Yet, MD-LPD placentas supported fetal growth to a level similar to that of NPD placentas.

In this study, paternal LPD significantly increased fetal weight, similar to previous observations by our group [18]. We also observed a shift in the distribution of LPD fetal weight, with 22% of fetuses exceeding the 90th centile for NPD weights. There is growing evidence in humans, that LGA offspring are at an increased risk of developing diabetes [28], obesity [29] and even some cancers [30]. Previous studies have found that a maternal low-protein diet in mice during the pre-conceptional period can increase fetal weight [27,31]. However, studies investigating the impact of paternal diet have had conflicting results. Paternal undernutrition (diet restricted to 70% of control intake) in mice has been found to reduce offspring weight, cause dyslipidaemia and alter adipose accumulation [32]. A similar finding was observed in mice with diet-induced paternal obesity, where both placental and fetal weights were reduced at term [33]. However, a separate paternal obesity mouse model found this dietary regime had no impact on fetal or placental weights [34], and another found that paternal diet-induced obesity resulted in heavier neonates which demonstrated impaired glucose tolerance and insulin resistance in later life [35]. Previous work investigating the physiology of males fed LPD and MD-LPD has demonstrated that there is no weight change in males fed LPD, however males fed MD-LPD exhibited increased weight gain and adiposity compared to NPD [20]. We have shown, in the current study, that the fetuses sired by these MD-LPD males had reduced weights compared to LPD and demonstrated a significantly reduced proportion of fetuses below the 90th centile. Interestingly, this data suggests that methyl-donor vitamin/mineral supplementation may prevent an LPD-associated overgrowth. Paternal folate deficiency has been shown to reduce fetal weight in rats [36], and has been associated with fetal and placental malformations in mice [21]. Conversely, supplementation of a restricted paternal diet with vitamins and antioxidants (including folate, vitamin C and vitamin E), in mice, was found to negate the offspring growth restriction observed with paternal undernourishment [32]. In humans, Pauwels *et al*. utilised detailed food diaries to calculate methyl-donor intake in men and determine associations between intake of methyl-donors (e.g. methionine, choline and betaine) and birth weight [37]. They found that different methyl-donors may correlate with changes in birth weight; with betaine and methionine demonstrating a negative association and choline a positive [37]. From these studies, it can be inferred that paternal micro/macronutrient balance is an important factor that must be considered when assessing reproductive outcomes. However, the males fed MD-LPD were previously found to have an increased weight gain and increased serum saturated fatty acids [20], which could suggest these males have a different metabolic profile to LPD and NPD. Therefore, it is not possible to discriminate between the programming effects of the methyl donors alone from those that may occur in response to the increased adiposity in MD-LPD males. Obesity in males is associated with a myriad of fertility related issues, such as impaired sperm motility, count and morphology, and has been associated with alterations in early fetal development [34,38,39]. Consequently, it must be taken into consideration that the MD-LPD associated fetal and placental changes observed in this study may be related to a change in paternal metabolic environment rather than a simple addition of methyl donors to a sub-optimal diet.

The placenta, being the organ dictating fetal weight, would be expected to follow the same growth distribution as its fetus. However, we did not find this to be true in this study. LPD and MD-LPD placentas had significantly reduced placental weights and LPD placental weight distributions did not follow the same pattern as the respective fetal weight distributions. Thus, in response to paternal LPD, the overgrown fetus is being supported by a placenta of reduced weight. This could suggest that a paternal LPD produces a more efficient placenta, reflected by the increased fetal:placental ratio. MD-LPD placentas, whilst demonstrating a significantly reduced weight, compared to NPD placentas, did not reflect the fetal:placental relationships of the LPD group. This could suggest that the absence of fetal overgrowth in this supplemented group was due to a change in the placental transport flux. However, fetal:placental ratios as a proxy for placental efficiency are only at best an estimation [40]. To fully answer how placental transport is impacted by paternal diet the expression and function of key transporters should be examined. Animal studies examining the influence of maternal under-nutrition on placental transport have revealed conflicting results. A low-protein diet in pregnant rats was found to reduce placental transport capacity, specifically involving a reduction in sodium-coupled amino acid transport, leading to growth restricted fetuses [41,42]. Further maternal under-nourishment studies in the rat found that the placental expression of glucose and amino acid transporters (SLC2A3, SLC38A2 and SLC38A3) was reduced, yet SLC38A4 was found to increase in response to a restricted diet [43]. Alternatively, studies investigating maternal undernutrition in mice have hypothesised a compensatory transport response, in that a restricted diet increases placental expression of *Slc2a1* and *Slc38a2* and placental transport of neutral amino acids at term, however these studies still observed fetal growth restriction [44,45]. We have previously shown that our paternal low protein diet increased the expression of placental mRNA expression of glucose transporters (*Slc2a1, Slc2a4*), amino acid transporters (*Slc38a2, Slc38a4*) and the calcium transporters, *Atp2b1* [18]. There is further evidence suggesting paternal high-fat diet can impact placental mRNA expression *Slc6a19* in a sex-specific manner, with females more affected that than males [46]. This raises an important caveat of placental investigations, that the sex of the fetus, and thus the placenta, can impact its response to sub-optimal conditions. Sex of the placenta is a crucial regulator of its transport and function [47], and male offspring have been found to have significantly poorer outcomes in pregnancies complicated by growth restriction [48] It was unfortunately not possible in our current study to investigate the fetal sex impact on response to poor-paternal diet.

The assessment of the placental phenotype conducted in this study revealed a proportional reduction of the junctional zone of LPD placentas, compared to both NPD and MD-LPD. The full functions of the junctional zone are not completely understood; however, there is evidence to suggest this zone acts as a site of energy reserves for the placenta and undertakes crucial endocrine functions; that are essential for fetal development as well as stimulating essential pregnancy-related adaptations to the maternal cardiovascular, immune and metabolic systems [[49], [50], [51]]. Whilst there are very few studies examining placental layer structure in relation to paternal diet, junctional zone abnormalities have been identified in placentas from undernourished mothers [44,[52], [53], [54]]. In a mouse model of sustained maternal protein restriction, the junctional zone area was found to be significantly reduced throughout gestation [54]. This study attributed the junctional size reduction to a loss of glycogen trophoblast (GlyT) cells [54]. In our current study the LPD-associated reduction in junctional zone proportion was not associated with a reduction in glycogen staining. Conversely, whilst MD-LPD placentas did not show any proportional junctional size differences compared to NPD, they exhibited a significant reduction in junctional-specific glycogen staining. The PAS staining used in the present study is commonly utilised to identify glycoproteins and has been used to highlight the location of GlyT [49]. These trophoblasts are proposed to act as energy reserves for the placenta, with the glycogen stores depleting as the fetal growth becomes exponential nearer term [49,55]. The decreased abundance of PAS positively stained cells in the MD-LPD placental junctional zone, suggests a loss of glycogen. However this would require assessment of the presence/absence of key receptors on glycogen cells and enzymes associated with glycogenolysis, such as glucose 6-phosphatase and glucagon, to affirm this speculation. Glycogen trophoblast cells have been found to have increased glucagon expression towards term, as it becomes necessary to utilise glycogen stores as glucose to support exponential fetal growth in late gestation [49]. Aberrations in placental glycogen storage and the reduction of GlyT numbers have been linked to a reduced fetal growth in both animal models and humans [56,57]. Previous studies have observed a reduced E15.5 placental glycogen content when maternal choline is supplemented throughout gestation in mice [58]. However by the end of gestation this was no longer apparent, with a higher glycogen content found as choline supplementation increased. The dynamic changes of glycogen content across gestation highlight the imperative need to assess whether there is an impairment of glycogen accumulation in MD-LPD placentas prior to E17.5 that altered histological glycogen abundance noted in our study, however this would require further detailed analysis of earlier gestational time-points as well as investigation of key genes involved in glycogen storage in the placenta, such as glycogen synthase. Furthermore, there was no significant difference in total placental glycogen content in this study. This could be due to the specific junctional location of glycogen, and in examining a mix of junctional and labyrinth zones any proportional differences were removed and thus the global placental assay may not be revealing the finer details. A more detailed investigation of this placental zone is required to fully understand the paternal diets relationship with GlyT and glycogen storage in the placenta.

MD-LPD placentas also demonstrated an increased whole placental area and junctional zone area compared LPD placentas, and the chorionic plate area was significantly greater than that of both NPD and LPD placentas. This suggests a disproportionate increase in layer composition in placentas from MD-LPD fathers, due to the labyrinth zone remaining similar to NPD. The chorionic plate plays a key role in the formation of the labyrinth layer [59]. The early chorionic ectoderm (pre-E9.0) has been found to be crucial in supporting labyrinth layer development with the abundance of trophoblast-stem cell progenitors [60], thus a change in chorion layer area could suggest that paternal methyl-donor supplementation has the potential to influence the lineage programming of the placenta. Under normal conditions the chorion area decreases as the labyrinth area increases, from approximately E12.5 [52,60]. It is unclear in this study why MD-LPD placentas demonstrate an expanded chorionic plate area yet no change in labyrinth area, to fully answer this an earlier time-point in placental development would need to be examined in future studies. The finding of increased total placental area but no change in proportions, alongside a reduction in placental weight suggest MD-LPD placentas had an altered shape. A more domed shape, with a possible reduction in placental circumference could account for these findings. Studies investigating SGA babies in humans have found evidence that a reduced placental diameter and smaller placental mass are associated with a reduced fetal weight [61]. This leads to an explanation for the observed fetal weight reduction in the MD-LPD group, however it cannot be purely size-related as these placentas had similar weights to the LPD placentas that produced overgrown fetuses. It is possible that there are more functional mechanisms at play within the placenta, potentially influencing placental transport, with paternal diet altering the synchronisation between placenta and fetus. Furthermore, the results reported in our study are not absolute volume measurements of placental areas, and thus cannot ascertain any firm conclusions regarding placental structure. A more robust analysis of the specific cell types and defined spaces (i.e. maternal blood space, fetal capillary areas etc.) would better define the influence of sub-optimal and supplemented paternal diets on placental structural.

To further determine placental adaptations in relation to the LPD-associated increased fetal growth, we assessed placental metabolism, investigating the FFA and cholesterol content. We found a reduced placental FFA concentration in LPD placentas compared to NPD and MD-LPD. The placenta regulates FFA delivery to the fetus, thus a lower concentration of FFA present in E17.5 placentas suggests reduced delivery. However, as the LPD fetal weights were elevated above NPD weights, it could be possible that there has been an accelerated fetal growth prior to this time point or that the growth is not dependent on FFA availability. In models of maternal nutrition, the reduced levels of FFAs in the diet have been associated with neurological development and metabolic disorders in the offspring [62,63]. Yet, our assessment of placental FFA content does not factor into account the possibility of altered FFA transport rates or metabolism. Modelling of the placental fatty acid transfer across the placenta by Perazzolo *et al*. suggested that fatty acid metabolism is the rate-determining factor for maternal FFA uptake, not membrane transport [64]. However, the majority of studies observing dysregulated placental transport of FFAs has been conducted in models of maternal over-nutrition/obesity [65]. There is evidence suggesting that placental FFA metabolism is influenced by maternal dietary deficiency, with a maternal vitamin-D deficiency reducing FFA levels in the placenta alongside a reduced placental weight [66]. Both FFA metabolism and transport would require examination to reveal details as to why LPD placentas demonstrated a higher FFA content in the current study. However, it is worth considering that as our paternal diet model cannot influence the availability of FFA in the mother, as these dams are maintained on identical standard chow diets, there is a possibility that the reduced placental FFA concentration is due to a potential programming of the placenta that is driven by the paternal diet. These changes, therefore, may be directed by differential gene expression in the placenta.

Paternal diet can influence the epigenetic status of his sperm [19,67], and in turn sperm epigenetic modifications have been found to influence early embryo cell-lineage determination and placental gene expression [34,68,69]. In this study, we examined the expression of genes involved in maintenance and modification of epigenetic signatures, as well as key paternally-expressed imprinted genes. We found that paternal MD-LPD reduced placental expression of *Dnmt3b*. Dnmt3b, in partnership with Dnmt3a, is responsible for the establishment of genomic DNA methylation patterns in the early placenta following implantation [[70], [71], [72]]. Our results suggest that an excess of methyl-donor supplements in the paternal diet may be able to alter the epigenetic status of the placenta. However, alterations in DNA methyltransferase levels are not essential for changes to global methylation [73], supported by the lack of changes in global placental methylation in this study. This area requires further study to fully understand the role of paternal diet-mediated control of placental methylation and epigenetic status.

In conclusion, this study revealed that paternal diet can direct fetal and placental weights, yet the influence of paternal LPD on fetal and placental development cannot be corrected by the simple addition of methyl-donor supplements. When taken together the findings for how paternal LPD impacts fetal and placental development suggests an uncoupling between placenta and fetus, in that the gross structure and gene expression profile mirrors that of NPD, yet LPD-associated fetal overgrowth was still observed. Furthermore, the supplementation of a poor diet with methyl-donors was not a ‘quick-fix’, as the placental morphology and gene expression was altered differentially to that seen in LPD placentas. These observations highlight the important need to understand how paternal sub-optimal diets influence the placenta and the impacts on fetal development, and further highlights that dietary supplementation in expecting fathers requires more in-depth studies to elucidate the underlying mechanisms that may alter placental development and function and impact the programming of future generations.

## Funding

This work was supported by an Aston Research Centre for Healthy Ageing fellowship awarded to AJW, a Society for Reproduction and Fertility Academic scholarship awarded to AJW and by funding from the 10.13039/501100000268BBSRC under grant BB/R003556/1 awarded to AJW.

## Declaration of competing interest

The authors declare that they have no known competing financial interests or personal relationships that could have appeared to influence the work reported in this paper.
