# Supplementary material for: Paternal low protein diet and the supplementation of methyl-donors impact fetal growth and placental development in mice
Source: Placenta. 2021 Jan 1;103:124–33. doi: 10.1016/j.placenta.2020.10.020 (PMC7907633; doi:10.1016/j.placenta.2020.10.020)
Supplement: Multimedia component 1 [file mmc1.docx]

**SUPPLEMENTARY FILE**

Supplementary Table 1: Compositions of the male diets

| **Dietary Supplement** | **NPD (g/kg)** | **LPD (g/kg)** | **MD-LPD (g/kg)** |
| --- | --- | --- | --- |
| Casein | 18.0 | 9.0 | 9.0 |
| Corn Oil | 10.0 | 10.0 | 10.0 |
| Starch Maize | 42.5 | 48.5 | 48.5 |
| Cellulose | 5.0 | 5.0 | 5.0 |
| Sucrose | 21.3 | 24.3 | 24.3 |
| Vitamins (AIN76) | 0.5 | 0.5 | 0.5 |
| Minerals (AIN76) | 2.0 | 2.0 | 2.0 |
| Choline Chloride | 0.2 | 0.2 | 0.7 |
| D,L-Methionine | 0.5 | 0.5 | 1.25 |
| Betaine | - | - | 1.5 |
| Folic Acid | - | - | 0.0015 |
| Vitamin B12 | - | - | 0.00015 |

Supplementary Table 2: Information on primer sequences for gene expression RT-qPCR

| **Gene Name** | **Gene Symbol** | **Accession number** | **Forward Primer** | **Reverse Primer** | **Amplicon length** |
| --- | --- | --- | --- | --- | --- |
| *TATA-Box Binding Protein* | *Tbp* | NM_013684.3 | GGGAGAATCATGGACCAGAA | GATGGGAATTCCAGGAGTCA | 90 bp |
| *Tubulin a* | *Tuba* | NM_011653 | CTGGAACCCACGGTCATC | GTGGCCACGAGCATAGTTATT | 114 bp |
| *DNA Methyltransferase 1* | *Dnmt1* | NM_010066.3 | GCTACCAGTGCACCTTTGGT | ATGATGGCCCTCCTTCGT | 73 bp |
| *DNA Methyltransferase 3 alpha* | *Dnmt3a* | NM_007872.4 | ACACAGGGCCCGTTACTTCT | TCACAGTGGATGCCAAAGG | 70 bp |
| *DNA Methyltransferase 3 beta* | *Dnmt3b* | NM_001003961.4^[[1]](#footnote-1)^ | GCCTGCAAGACTTCTTCACTACT | GGTACAACTTGGGTGGCTCA | 63 bp |
| *DNA Methyltransferase 3 like* | *Dnmt3l* | NM_019448.3^*^ | AACCGACGGAGCATTGAA | CCGAGTGTACACCTGGAGAGT | 60 bp |
| *Histone Deacetylase 1* | *Hdac1* | NM_008228.2 | TGGTCTCTACCGAAAAATGGAG | TCATCACTGTGGTACTTGGTCA | 78 bp |
| *Histone Deacetylase 2* | *Hdac2* | NM_008229.2 | TGCTGTTCATGAAGACAGTGG | TTTGTCTGATGCTCGAATGG | 76 bp |
| *Lysine (K)-Specific Demethylase 3A* | *Kdm3a* | NM_173001.3 | TCTGGGATGGATTTGAAGATG | AAACCTGGAAGGCATCATGT | 125 bp |
| *Methyltransferase Like 3* | *Mettl3* | NM_019721.2 | TAAACCACGGGAAGGAACAC | TTATGACTGGTGGAACGAACC | 112 bp |
| *Methyltransferase Like 14* | *Mettl14* | NM_201638.2 | GCAGCACCTCGGTCATTTAT | TCTTCTGTAACCCCACTTTCG | 93 bp |
| *Insulin-like growth factor 2* | *Igf2* | NM_010514.3 | CGCTTCAGTTTGTCTGTTCG | GCAGCACTCTTCCACGATG | 95 bp |
| *Small Nuclear Ribonucleoprotein Polypeptide N* | *Snrpn* | NM_00108296.1 | GACACCAAGAGGTGGTTAAAGC | GGATGGGTCCTGTTTTCCTT | 71 bp |
| *Mesoderm-specific transcript homolog protein* | *Mest* | NM_008590.1 | GATAATGCGGCCATGGTG | AGGTACGCAGCCAGCAAG | 98 bp |

1. *Accession number identifies variant 1, however primer designed to detect all variants* [↑](#footnote-ref-1)
